# Hashtagging Resistance: Media, Authority, and Women's Subjectivity in Post-Mahsa Iran

**DOI:** 10.1177/10778012251379446

**Published:** 2025-09-24

**Authors:** Arash Beidollahkhani, Mahdiyeh Ghorashi

**Affiliations:** 1 Global Development Institute, 5292 University of Manchester, Manchester, UK; 2 Department of Political Science, 48440 Ferdowsi University of Mashhad, Mashhad, Iran

**Keywords:** subjectivity, authority, Hijab, Mahsa Amini, digital resistance

## Abstract

This paper explores the intersections of media, authority, and women's subjectivity in post-Mahsa Iran through Lacanian psychoanalysis. We examine digital activism on X (formerly Twitter) following Mahsa Amini's death in 2022, focusing on how women resist state-imposed hijab laws by deploying hashtags as tools of symbolic disruption. Using a mixed-methods approach, we analyze 20 hijab-related hashtags from 2018 to 2023 to reveal how these campaigns express resistance and shape psychological subjectification. Our findings underscore women's resistance as both a site of subjectivity and political agency, illustrating how digital activism fosters feminist identity, symbolic defiance, and subject formation under authoritarian rule.

## Introduction

Since the 1979 Islamic Revolution, the hijab has remained a central ideological concern for the Islamic Republic of Iran. Over the past four decades, it has evolved from a religious and jurisprudential obligation into a politicized symbol, deeply tied to state authority and social control. Successive governments have promoted hijab not only as a marker of religious piety but also as an indicator of women's chastity and national morality. Consequently, state-imposed dress codes for women have endured ongoing public objections since the revolution's earliest years.

Although the law mandating hijab was formally instituted in 1984, women began resisting it from the outset. What began as informal disobedience grew into open criticism and protest. Over time, the state institutionalized and enforced varying interpretations of hijab, leading to what is commonly referred to as “bad hijab,” a term used to describe noncompliant or loosely worn hijab styles (Bayat & Hodges, 2022). This shift marked a transition from total rejection of hijab to a more symbolic, negotiated form of resistance. In doing so, the hijab became a social and political flashpoint, no longer confined to the private sphere but deeply embedded in public discourse and governance.

Framing hijab enforcement as a matter of national security and moral order, the state introduced a series of formal initiatives beginning in 2007 under the banner of promoting “social security.” These efforts, typically launched during summer and winter seasons, were aimed at policing women's dress in public spaces. Critics argued that such policies extended far beyond religious enforcement and served as mechanisms for defending the state's ideological boundaries and political authority. The hijab thus became a visible sign of compliance with, or resistance to, state power. As a result, noncompliance was criminalized and interpreted as an antivalue behavior—prompting legal consequences and surveillance (Teimouri, 2024).

This escalation of control provoked a wave of civil disobedience among Iranian women. Acts of resistance against mandatory hijab have appeared intermittently since the early years of the Islamic Republic, but they intensified notably in recent years. In 2018, journalist and activist Masih Alinejad^1^ launched the White Wednesdays campaign, encouraging Iranian women to share videos of themselves removing their hijab in public (Alinejad, 2018). This campaign, which employed hashtags and social media platforms as tools of mobilization, marked a significant shift from isolated protest to a more organized, networked form of digital activism.

The campaign laid the groundwork for broader resistance that culminated in the nationwide protests following the death of Mahsa Amini in September 2022. Amini's death in custody after being arrested by the morality police sparked what became known as the Zhina Movement, a massive, youth-led uprising challenging not only the mandatory hijab but also the legitimacy of the political system itself. Women's visibility in public spaces without hijab became a powerful symbolic act, complemented by widespread nightly demonstrations and social media campaigns (Shaban, 2022). Though the movement did not engage all sectors of society, it galvanized a significant portion of the population and amplified calls to abolish compulsory veiling laws (Khatam, 2023).

Importantly, this is not the first time Iranian women have resisted state-imposed dress codes. During the reign of Reza Shah in the 1930s, the forced unveiling policy also sparked backlash, though protests were fragmented and lacked organization (Basmechi, 2019; Basmechi et al., 2022). In contrast, today's resistance has grown out of decades of struggle and now operates within an interconnected, digitally empowered environment. Despite sustained repression, Iranian women have used their bodies—and increasingly, digital tools—as platforms for asserting autonomy, challenging gendered control, and redefining the meaning of hijab in contemporary Iran.

Against this historical and political backdrop, this paper investigates how Iranian women's resistance has entered a new phase shaped by digital technologies and platform-based activism. Focusing on the years 2018–2023, the study analyzes how hashtag campaigns on Persian X (Twitter) have become central tools for articulating feminist dissent and reclaiming symbolic power under conditions of authoritarian surveillance. While the enforcement of hijab laws continues to shape women's public and private lives, digital spaces now offer new arenas for protest, visibility, and identity formation. This paper integrates empirical analysis of hashtag trends with Lacanian psychoanalysis to explore how acts of digital resistance, particularly through recurring, politicized hashtags, reflect deeper processes of subjectivity, symbolic rupture, and subversion. In doing so, it bridges the empirical dynamics of digital feminist movements with a theoretical framework that explains how women confront, negotiate, and reimagine their roles within the state's ideological apparatus.

In the contemporary digital era, the pervasive accessibility of the internet has fundamentally transformed the landscape of women's protests. Resistance strategies have shifted from traditional offline acts of civil disobedience to encompass symbolic protests and digital activism, signifying a critical evolution in both the medium and the messaging of feminist movements (Afary & Anderson, 2023). This transformation underscores the interplay between technological affordances and the reconfiguration of gendered political expression within digital spaces.

The historical content and themes of women's protests, particularly those concerning the hijab, have achieved global visibility through hashtags that frequently develop into international trends (Tufekci, 2017). This digital amplification empowers women to orchestrate resistance movements beyond the direct oversight of governmental authorities. Protests initiated within the internet, a term encompassing digital platforms and their associated networks, can produce tangible real-world outcomes, eliciting political responses from diverse groups (Castells, 2001). X (Twitter) emerges as a pivotal platform in this context, facilitating the dissemination and coordination of feminist activism (Siemon et al., 2024). However, this reliance on digital spaces introduces vulnerabilities, such as risks of surveillance and co-optation, necessitating a critical examination of the internet's role in contemporary resistance movements (Dal & Nisbet, 2022).

Since women's subjectivity, defined as a psychoanalytically informed process of self-articulation within symbolic structures (Kruks, 1992), is central to the discourse of resistance against the state's ideological control over hijab, the paper utilizes Lacan's ideas as the theoretical framework to explore women's inclination toward subjectification in X hashtags (Day, 2013).

Resistance, as conceptualized in this study, refers to digital and symbolic acts that challenge state-sanctioned norms. It includes civil disobedience (e.g., appearing unveiled in public), symbolic performativity (e.g., standing on platforms with a hijab—scarf—in hand), and networked digital activism (e.g., repeated use of counter-hegemonic hashtags). The focus is primarily on symbolic resistance through digital media, though it intersects with offline actions and civil resistance (Elmore, 2024; Richer, 2024). This triadic definition is crucial given the blurred boundaries between online and offline activism in Iran's current sociopolitical landscape.

Despite growing scholarly attention to Iranian women's resistance, few studies have connected digital activism to psychoanalytic theories of subject formation. Existing literature often emphasizes political mobilization or sociological dynamics, but the psycho-symbolic dimensions of women's protest, especially in how digital language reshapes identity, remain underexplored (Ahmadi, 2024; Batyari, 2025; Chubin, 2024; Izadi & Dryden, 2024; Pourmokhtari, 2022; Sameh, 2019; Seddighi, 2023; Sigurdardottir et al., 2024; Tafakori, 2021; Tohidi & Daneshpour, 2025; Walsh, 2024).

This study addresses that gap by applying Lacanian psychoanalysis to analyze digital feminist resistance on Persian X (Twitter). Specifically, it examines how women's subjectivity is expressed, negotiated, and transformed through the repeated use of politicized hashtags. This approach offers a novel contribution by showing how symbolic subversion in digital platforms serves both psychological and political functions in authoritarian regimes. By tracing hashtag trends from 2018 to 2023, we map the evolution of women's discursive resistance and its alignment with deeper processes of subjectification.

## Psychoanalytic-Political Framework

### Conflict Between Lacanian Subject and Master Discourse

According to Jacques Lacan, identity is not fixed at birth but emerges continuously through interactions with the symbolic structures that shape human life—language, law, ideology, and social norms (Felluga, 20023; Hendrix, 2019). These symbolic domains provide the frameworks through which individuals attempt to articulate themselves as subjects. Within each domain, Lacan argues, dominant ideological forces—what he calls “master discourses”—work to define and constrain identity (Lacan, 1966, 1954/1988). These discourses impose norms and expectations that aim to stabilize social order, often suppressing individual agency and desire in the process (Fink, 1995; Laclau & Mouffe, 1985). In Lacan's schema, the subject enters into symbolic life upon learning the word “I,” a pivotal moment in which one becomes recognizable within a shared linguistic and ideological order. However, this entry is marked by a fundamental conflict: the subject's pursuit of self-definition inevitably clashes with the constraints imposed by the master discourse (Lacan, 1955/1991). The master discourse, in its authoritarian function, does not seek mutual understanding or knowledge but rather seeks to reproduce itself by compelling submission to its own norms—what Lacan refers to as the law or the “Name-of-the-Father” (Žižek, 1989). In this structure, the individual's own desire is subordinated to the will of the master, positioning the subject as an object within the symbolic order. This creates a psychic and political tension: individuals must negotiate their emerging subjectivity within systems that continually attempt to fix and regulate identity. Becoming a subject, in Lacanian terms, thus requires a form of symbolic defiance—a refusal to fully identify with the dominant order and an effort to assert one's own desire, often in conflict with the prevailing ideological structures (Gallop, 1982; Homer, 2006). These dynamics are especially visible in politicized environments where state power seeks to define norms, rituals, and identities, such as the case of hijab enforcement in Iran.

Building on this foundational tension between the subject and the master discourse, Lacanian psychoanalysis becomes particularly useful for analyzing resistance in digital political environments, especially under authoritarian regimes. The symbolic structures that Lacan describes are not confined to traditional institutions like law or family; they are also encoded in state ideology, media narratives, and cultural norms. In Iran, the hijab operates as a powerful ideological signifier, one that encapsulates the moral and political authority of the Islamic Republic. When women resist this signifier, particularly through digital activism, they do more than oppose a dress code; they symbolically reject the master discourse itself. This creates a rupture in the symbolic order, opening up space for new subjectivities to emerge. Social media platforms, especially X (Twitter), offer fertile ground for these disruptions, functioning as arenas where marginalized voices articulate new forms of identity and resistance. This paper applies Lacanian theory to these digital practices, tracing how hashtags serve as tools for subjectification and symbolic rearticulation.

In the context of digital activism, Lacanian psychoanalysis offers a valuable lens to understand how subjects emerge through confrontation with dominant discourses (Johnston, 2018). Social media platforms, particularly X (Twitter), function as symbolic arenas where individuals, previously relegated to object positions within state discourse, begin to articulate themselves as subjects. The state's imposition of the hijab operates as what Lacan might call a “master signifier”: a fixed, nonnegotiable symbol that anchors the ideological structure of the Islamic Republic. In this framework, women's resistance, especially via digital platforms, constitutes a rupture in the symbolic order (Chapman, 2016). Hashtags like #womanlifefreedom and #noToMandatoryHijab are not merely political slogans but represent the articulation of desire that escapes the state's normative gaze, allowing women to shift from objectified roles (the “Other” in Lacanian terms) to self-articulated subjects.

Moreover, X (Twitter) activism represents a new form of what Lacan describes as a refusal of the Other's desire. By rejecting the imposed identity (e.g., the “modest” woman under state definitions), Iranian women are not only challenging political authority but also enacting a psycho-social transformation. The creation and repetition of hashtags represent a “signifying chain” where the subject attempts to reinscribe herself in the symbolic order on her own terms. This realignment of subjectivity through digital resistance is central to the political meaning of activism in authoritarian settings like Iran.

In this study, subjectivity refers to the psychoanalytic process through which women come to recognize themselves as agents within a symbolic order. In Lacanian terms, this occurs when the subject enters language and begins to formulate the “I” in opposition to the Master discourse (Sheikh, 2017). Subjectivity here is not merely individual agency or identity but is understood as an unstable, discursively constructed position negotiated in relation to dominant norms, specifically, the state's discourse on gender and morality (Lacan, 1954/1988).

By contrast, subjectification is used to describe the process by which individuals become subjects within or against these symbolic structures (Lacan, 1955/1991). In the context of hijab protests, subjectification involves Iranian women refusing to be positioned as passive recipients of the state's ideological scripts, instead asserting their autonomy by producing counter-discourses—often through digital media. Hashtags such as #IAmAFReeWoman or #NoToMandatoryHijab are interpreted as acts of subjectification, where the symbolic space of X (Twitter) becomes a battleground for redefining the female subject.

In this study, X is examined not merely as a communication tool but as a symbolic text, a digital arena in which marginalized groups challenge dominant ideological frameworks. While traditional political science has often overlooked the discursive potential of social media platforms, recent scholarship underscores their critical role in shaping political subjectivity, particularly for actors excluded from official narratives (Boulianne, 2015; Breuer, 2016). In the Iranian context, where the hijab functions as a central signifier of the state's moral and political authority, any deviation from its prescribed form becomes a powerful act of symbolic resistance. Hijab is not just clothing but a language of state ideology, an emblem through which the Islamic Republic articulates its vision of gender, morality, and control (Ahmed, 2011; Gould, 2014). As such, when women reject or reinterpret the hijab especially in visible, networked spaces, they disrupt the master discourse that seeks to define them.

This disruption is enacted through the use of politicized hashtags, which serve as acts of self-naming and vehicles of subjectification. These hashtags do not simply communicate dissent; they constitute it. By repeatedly engaging in hashtag activism such as #NoToMandatoryHijab and #WomanLifeFreedom, women in Iran assert their symbolic and political agency within a heavily regulated discursive field. From a Lacanian perspective, these acts mark the subject's refusal to be positioned solely as the Other within the state's symbolic order. Instead, they represent an effort to inscribe the “I” into public visibility on one's own terms. The circulation and accumulation of these hashtags thus reflect more than a protest trend; they constitute a psycho-symbolic process through which new subjectivities emerge in direct defiance of the dominant ideological framework.

Two additional Lacanian concepts are relevant for understanding the dynamics of feminist digital protest: speech act and jouissance. In Lacan's framework, a speech act is not merely communicative but performative; it marks the subject's active entry into symbolic discourse, often as a rupture from imposed silence or objecthood. In the Iranian context, hashtags such as #IAmAFReeWoman or #HijabIsNotMyChoice function as speech acts, where women symbolically reassert their subjectivity against the state's ideological interpellation.

The concept of jouissance refers to a form of excessive or transgressive enjoyment that emerges when desire exceeds the bounds of symbolic law. It is affectively intense, sometimes painful, and often tied to acts of resistance that defy moral or ideological containment. In hashtag activism, jouissance appears in the form of emotionally charged statements that blend grief, irony, rage, and euphoria—revealing a libidinal investment that cannot be reduced to rational dissent alone.

## Online and Offline Activism: The Interplay of Campaigns and Hashtags in Driving Collective Action

Recent advances in information and communication technologies have redefined how citizens engage with politics, offering alternative arenas for resistance, especially in contexts where traditional public expression is constrained (Bimber et al., 2014; Boulianne, 2020; Boulianne et al., 2023; Jungherr et al., 2020; Novelli & Sandri, 2024; Oser et al., 2022; Zhuravskaya et al., 2020).

In authoritarian societies like Iran, digital platforms offer a mediated public sphere where previously suppressed identities and voices can gain visibility. These spaces do not merely supplement offline political participation; they shape entirely new forms of collective action, enabling users to circumvent state-controlled institutions and narrate their own dissenting subjectivities.

Digital campaigns and hashtags have become central to the architecture of contemporary social movements, serving as organizing tools, symbolic markers, and catalysts for transnational solidarity. The concept of “connective action” highlights how digital platforms facilitate personalized content sharing, enabling decentralized mobilization without traditional hierarchical structures (Bennett & Segerberg, 2013). Hashtags like #BlackLivesMatter and #MeToo exemplify how digital symbols can unify diverse individuals around shared grievances, fostering a sense of collective identity and purpose (Jackson et al., 2020). These digital markers not only coordinate actions but also create discursive spaces where marginalized voices can challenge dominant narratives, contributing to the formation of counterpublics (Fraser, 1990). Moreover, social media platforms have been instrumental in the rapid dissemination of information, as seen in movements like Occupy Wall Street, where X (Twitter) facilitated real-time coordination and global awareness (Gargiulo et al., 2015). However, the reliance on digital platforms also presents challenges, including issues of representation and the potential for movements to be co-opted or diluted over time (Garza, 2020).

In the Iranian context, this global phenomenon takes on particular urgency. Hashtags have become essential instruments for resisting ideological repression, asserting agency, and confronting the symbolic power of the state in highly censored political conditions.

Unlike earlier waves of digital engagement that emphasized online mobilization as separate from real-world action, current scholarship points to the interplay between online expression and offline resistance (Smith et al., 2023; Van Laer & Van Aelst, 2009). The architecture of platforms such as X (Twitter) encourages immediacy, virality, and discursive confrontation, creating conditions where marginalized groups can contest dominant ideologies and generate counter-hegemonic narratives in real time. In this regard, X functions not only as a communication tool but also as an infrastructure of symbolic disruption, what scholars have described as a “movement-generating medium” (Duncombe, 2019).

What distinguishes digital resistance in Iran is its strategic use of platform-specific affordances to subvert the ideological reach of the state. Despite being filtered, X (Twitter) remains highly influential among politically active users, feminism and women activists, and diasporic communities (Banerjee & Kankaria, 2022). Its continued relevance highlights how censorship can be negotiated through proxy servers and VPNs, allowing dissenting publics to persistently reenter the symbolic field (Wulf et al., 2022). These publics do not rely on centralized leadership but instead engage in connective action—loose, decentralized forms of engagement facilitated by personalized content circulation (Bennett & Segerberg, 2013).

In the Iranian context, the shift from offline to online activism has not merely mirrored traditional protest methods; it has fundamentally reshaped the architecture of resistance. Faced with decades of state suppression, legal restrictions, and surveillance of physical gatherings, activists increasingly turned to digital platforms as strategic extensions of the public sphere (Beidollahkhani, 2022). What once required public presence in streets and university campuses has been rearticulated through symbolic gestures—videos, hashtags, and coordinated posting—that replicate the performative power of offline protest while mitigating physical risk. Digital environments have thus become surrogate spaces for collective action, where movements are not only organized but also sustained, memorialized, and globalized (Baran & Stoltenberg, 2024). This transformation reflects a recalibration of activist strategy, enabling forms of civil disobedience that transcend geographic boundaries, evade immediate repression, and create transnational visibility for localized struggles (Mehta, 2024).

This shift has also transformed the nature of subject formation in political activism. The symbolic economy of X (Twitter), comprising keywords, metrics, retweets, and trending topics, offers a new semiotic system through which users articulate political presence (Leonel et al., 2024). The platform's algorithmic logics do not determine meaning but structure its circulation. In this way, political engagement on X becomes performative and recursive, allowing for the reinscription of alternative subjectivities that challenge the state's attempt to fix meaning through dominant signifiers (Cheng et al., 2023).

For Iranian women resisting the ideological weight of the hijab mandate, these digital structures offer more than technical tools; they provide symbolic entry points into the public sphere. This process is not without risk. Online resistance is constantly exposed to surveillance, harassment, and digital repression. Yet even within these constraints, the persistence of feminist expression in digital platforms signals a reconfiguration of agency. Rather than merely contesting visibility, activists reshape the terrain of visibility itself, expanding the symbolic boundaries of resistance beyond what is materially allowed within Iran.

## Research Design and Questions

This study examines how Iranian women use X (Twitter) as a symbolic space to contest the dominant state discourse on hijab and to articulate new feminist subjectivities. The research adopts a mixed-methods design, integrating both quantitative and qualitative strategies to analyze hashtag activism in the context of Iran's digital public sphere.

The research is guided by the following questions:

*Research Question 1:* How have Iranian women employed X hashtags between 2018 and 2023 to express resistance against the state-mandated hijab and broader systems of patriarchal control?
*Research Question 2:* How can Lacanian psychoanalysis, particularly its concepts of subjectivity, subjectification, and the master discourse, help us interpret the discursive construction of feminist resistance in digital platforms under authoritarian governance?

These questions situate the study at the intersection of media, psychoanalysis, and Middle Eastern gender politics. The selection of Lacanian theory is driven by its utility in explaining how individuals form subjectivities through language within power-laden symbolic structures. In authoritarian regimes like Iran, where the state attempts to monopolize symbolic authority, especially around gender and morality, digital activism becomes a space for discursive subversion. By applying Lacanian concepts, this study aims to illuminate the psychological and symbolic mechanisms through which women resist, reclaim identity, and redefine themselves in the digital sphere.

## Methodology

This study utilizes a mixed-methods approach that combines summative content analysis (quantifying hashtag usage over time) and conceptual content analysis (interpreting patterns of meaning within tweets) to analyze feminist hashtag activism on Persian X (Twitter). This approach allows for both macrolevel trend analysis and microlevel discursive interpretation.

The time frame of the analysis spans from March 2018 to March 2023, as it captures key phases in Iranian feminist digital activism, from the emergence of campaigns such as #WhiteWednesdays and #GirlsOfEnghelabStreet, to the pivotal moment of Mahsa Amini's death in September 2022 and the subsequent surge in digital protests. This 5-year period offers longitudinal insight into the evolution of resistance discourse.

A total of 20 hashtags were selected based on their thematic relevance, resonance in Persian X (Twitter), and linkage to major feminist campaigns or protest moments. The selection was purposive, informed by prior literature on Iranian digital activism, and refined through preliminary data exploration using the Keyhole analytics platform.

Data for this research was collected using Keyhole, a real-time social media monitoring and analytics tool designed to track hashtags, keywords, and user engagement across platforms. Keyhole provided tweet volume, engagement metrics (retweets, replies, likes), and time-series analytics highlighting peaks in hashtag usage. It also identified top content generators, such as activists and influencers, and offered geographic and demographic metadata when available.

This software has previously been used in social media research related to political communication and activism (e.g., Lenti et al., 2025; Mendelsohn et al., 2024). The use of Keyhole enabled longitudinal mapping of hashtag usage and insight into the actors and moments that shaped online resistance discourse (Keyhole, 2024).

Using Keyhole, we extracted a data set of approximately 1.2 million tweets and retweets related to the 20 selected hashtags, capturing tweet volumes, engagement rates, hashtag surges, and key contributors.

The conceptual coding process was primarily deductive, using Lacanian concepts drawn from the theoretical framework. Each code was defined with reference to specific linguistic and symbolic features. For example, “negation of the Other's desire” was identified through statements that overtly rejected imposed identity or moral roles (e.g., “I am not your honor” and “Hijab is not my choice”), while “master signifiers” were coded when a tweet repeated or contested fixed ideological terms like “modesty,” “chastity,” or “Islamic values.” Subjectivation was marked through self-declarative language that asserted presence, such as “I am a free woman.” A reflexive memoing process was employed by the author throughout coding to ensure consistency and transparency. While the coding was conducted by a single researcher, intercoder reliability was approximated through trial coding and feedback discussions with two peer reviewers familiar with Lacanian theory and Persian language, to ensure clarity and alignment with theoretical constructs. Furthermore, these analyses were conducted in both Persian (original tweets) and English (translated by the author, fluent in Persian), ensuring accurate cultural interpretation.

The dual use of summative and conceptual content analysis is justified by the study's dual goals, which aim to measure the digital visibility and temporal rhythm of hashtag campaigns in a quantitative manner while also interpreting the symbolic content and identity-formation processes embedded in these digital expressions in a qualitative context. Summative analysis allowed for identifying high-frequency hashtags and time-based peaks, while conceptual analysis illuminated how women resist the master discourse and construct alternative subjectivities online. This dual method aligns directly with Lacanian theory, which foregrounds the role of language and symbolic structures in identity formation. Hashtags are treated as “signifiers” in Lacanian terms, tools that both reflect and reshape subjectivity in a discursive field dominated by state-imposed norms.

Data on high-frequency content generators, such as Masih Alinejad, Iran International, and Tavaana,^2^ was incorporated to understand the networked dynamics of digital activism. While these individuals were not the focus of profile analysis, their prominent roles in hashtag diffusion helped contextualize how symbolic authority is challenged or redistributed in the digital field. To maintain ethical integrity, only publicly available metadata was used, and no personal data beyond account names was analyzed.

For conceptual content analysis, a stratified sample of 1,000 high-engagement tweets was selected from the larger data set, proportionally drawn across four key protest periods (2018–2023), major hashtag clusters, and political contexts. Tweets were coded for linguistic features, recurring motifs, and rhetorical structures aligned with Lacanian categories, using a deductive framework focused on three key constructs: (1) invocation or subversion of master signifiers (e.g., “honor” and “chastity”); (2) symbolic rupture, marked by irony, contradiction, or disidentification; and (3) negation of the Other's desire, in which tweets rejected imposed gendered subject positions. Approximately 45% of the coded tweets engaged with master signifiers, 30% reflected symbolic rupture, and 25% enacted subjective negation. While coding was conducted by the author(s), interpretive reliability was supported through iterative memo-writing, theoretical reflection, and consultation with two researchers trained in Lacanian discourse analysis and Persian-language digital activism. These procedures ensured that the selected tweet excerpts were not anecdotal but representative of broader psycho-discursive patterns within the data set.

The individuals identified by name in this study are well-known public figures—either celebrities or prominent social and political activists—whose real identities are widely recognized in Iranian society. Their inclusion is justified by their significant influence on public discourse and their large followings, which amplify the reach and impact of digital resistance campaigns. In sum, this methodological framework ensures analytical rigor, longitudinal depth, and theoretical alignment, making it well suited for examining symbolic resistance in authoritarian digital contexts.

## Quantitative Results

The tweets, retweets, quotes, opinions, and number of content publishers were analyzed over a 5-year period from March 2018 to March 2023. During this time, key digital campaigns such as Girls of Enghelab Street and White Wednesdays emerged, and the death of Mahsa Amini marked a major turning point in online feminist resistance.

The hashtag #WomanLifeFreedom recorded the highest frequency, with 568,416 tweets and 2,566,422 retweets. The most prominent sources of this hashtag between September 2022 and March 2023 were Ali Karimi,^3^ Masih Alinejad, Iran International,^4^ and Behnam Gholipour.^5^ Its Farsi translation ranked second, with 960,208 tweets and 208,637 retweets; Masih Alinejad was the top publisher of this version, generating 116,100 tweets.

The third most studied hashtag, #No to Mandatory Hijab, was tweeted 26,934 times and retweeted 38,852 times. The highest volume of tweets (*N* = 23.7 K), from June 2022 to March 2023, was generated by the Tavaana website. The hashtag #No to Hijab appeared in 17,818 tweets and 90,051 retweets, with Masih Alinejad emerging as the most influential publisher during June and July 2022, generating 111.6 K instances of this hashtag.

The hashtag #Girls of Enghelab Street ranked fifth in terms of frequency, with 5,462 tweets and 14,254 retweets recorded between August 2020 and February 2023. Key contributors included Masih Alinejad, Tavaana, Mostafa Tajzadeh,^6^ and the Manoto newsroom.^7^ The sixth most frequent hashtag was #No to Violence Against Women, tweeted 3,936 times and retweeted 6,038 times. An account labeled Marylyn, categorized as unpopular, was responsible for 2.1 K tweets using this hashtag from November 2018 to September 2022.

The slogan #Our Camera, Our Weapon reached a maximum publication frequency of 615.2 K, mainly through Masih Alinejad and Tavaana. It was tweeted 3,757 times and retweeted 40,904 times. Additionally, Masih Alinejad, Manoto newsroom, Iran International, and IranWire^8^ collectively generated #White Wednesdays 2.7 K times. This hashtag appeared most frequently during two specific intervals: March 2018 to November 2019 and October 2021 to March 2023, during which it was tweeted 3,062 times and retweeted 19,056 times.

The hashtag #Optional Hijab also showed notable activity between September 2020 and November 2022. However, its usage declined significantly between March 2021 and March 2022. It was tweeted 1,757 times and retweeted 3,833 times.

#Gender Equality was actively used throughout the study period, predominantly by an unpopular account named TorangeApp, in the form of quotes, opinions, and retweets. The hashtag was tweeted 1,372 times and retweeted 1,595 times.

The hashtag #Walking with No Hijab appeared 48.9 K times, mainly posted by Masih Alinejad. The highest tweet (*N* = 466) and retweet (*N* = 3,823) frequencies were recorded during two distinct intervals: March 2018 to November 2020, and September to November 2022.

Next, #Equality of Men and Women, mostly generated by the unpopular account Alireza, peaked in December 2018 and December 2020. It was tweeted 385 times and retweeted 189 times. The 13th most used hashtag, #No to Religious Government, was mainly promoted by the account Fariba, with 321 tweets and 382 retweets.

Other hashtags included #My Body, Woman's Body, #Woman Cannot Be Eradicated, and #Sneaky Freedoms, tweeted 265, 158, and 143 times, and retweeted 312, 401, and 421 times, respectively. These hashtags were mainly disseminated by unpopular accounts such as Farin Asmi, Shadi, and Rozhin Online, while Masih Alinejad was the most active user between September and November 2022, with a maximum frequency of 1.6 K tweets.

The hashtag #Brave Women was used 3.7 K times by UsabehFarsi and VOA, with appearances limited to February 2019, February 2021, and October 2022. #I Am a Free Woman gained popularity following Mahsa Amini's death, particularly during October and November 2022. This hashtag was generated 40 times by Reza Mohammadi, an unpopular account, and The Youth of Isfahan's Neighborhoods.

Finally, the hashtags #Hijab Is Not My Choice and #I Am Nobody's Honor were generated by two unpopular accounts, Irandokht and Pari, respectively. The former received intermittent attention throughout the study period, with increased frequency between March 2022 and March 2023. The latter appeared only during June 2020 (as retweets and opinions) and again in January 2022 and between October and November 2022.

Table 1 presents the frequency distribution of 20 selected hashtags related to Iran's anticompulsory hijab campaigns and broader feminist digital activism, collected from X (Twitter) between March 2018 and March 2023. These hashtags were chosen based on their thematic relevance, visibility in major protest campaigns, and engagement metrics across multiple time intervals. The table includes original tweet counts, retweet volumes, and identifies key publishers who significantly contributed to the dissemination of each hashtag. This quantitative overview provides a foundational understanding of the digital resonance and temporal dynamics of hashtag-based resistance in Iran. By highlighting variations in frequency and engagement, the table also offers insight into the role of individual influencers, news agencies, and grassroots participants in shaping the discursive field of feminist activism online. These metrics inform the subsequent qualitative analysis by identifying moments of peak activity and tracing how specific hashtags contributed to the construction of collective narratives in the post-Mahsa Amini era.

**Table 1.** Frequency Table of 20 Selected Hashtags on X (Twitter).

Hashtag	Tweets	Retweets	Impressions	Active Generators	Key Accounts	Peak Period	Engagement Type
Woman, life, freedom	568,416	2,566,422	12,608,483	348,808	Ali Karimi, Masih Alinejad	Sep 2022–Mar 2023	RT/opinion
Woman, life, freedom (Kurdish)	51,960	208,637	1,178,453	70,968	M. Alinejad	Sep 2022–Mar 2023	RT/opinion
No to hijab	17,818	90,051	773,920	24,244	M. Alinejad	Jun–Jul 2022	RT
Our camera, our weapon	3,757	40,904	851,378	18,081	M. Alinejad, Tavaana	Jun 2020– Jan 2023	RT
No to mandatory hijab	26,934	38,852	466,482	34,680	Tavaana	Jun 2022–Mar 2023	Opinion/RT
White Wednesdays	3,062	19,056	326,241	12,417	M. Alinejad, Manoto	Mar 2018–Mar 2023	RT
Girls of Enghelab street	5,462	14,254	282,257	12,980	M. Alinejad, Tavaana	Aug 2020–Feb 2023	Tweet/RT
No to violence against women	3,936	6,038	80,228	7,329	Marylyn (an unknown account)	Nov 2018–Sep 2022	Tweet/RT
Optional hijab	1,757	3,883	61,703	4,963	Maryam Karimbeigi	Sep 2020–Oct 2022	RT
Walking with no hijab	466	3,823	71,875	2,543	M. Alinejad	Mar 2018–Nov 2022	RT
Gender equality	1,372	1,595	21,291	2,344	Torange App	Sep 2020	RT
Brave women	123	744	5,714	836	Usabehfarsi, VOA	Feb 2019–Oct 2022	RT
Sneaky freedoms	143	421	6,140	627	M. Alinejad	Sep–Oct 2022	RT
Woman cannot be eradicated	158	401	4,912	541	Shadi (an unknown account), Rozhin Online	Aug–Sep 2020	Tweet/RT
No to religious government	321	382	2,970	522	Fariba (an unknown account)	Oct 2020–Jan 2023	RT/quote
My body, woman's body	265	312	12,685	609	Farin Asmi (an unknown account)	Jun 2018–Sep 2021	Tweet/RT
Equality of men and women	358	189	3,364	480	Alireza (an unknown account)	Dec 2018–Dec 2020	opinion/RT
I am a free woman	36	34	212	92	Reza Mohammadi (an unknown account) from the X youth group from Isfahan's neighborhoods	Oct–Nov 2022	RT/opinion
Hijab is not my choice	19	22	420	38	Irandokht (an unknown account)	Dec 2020–Nov 2022	RT/opinion

### Temporal Dynamics of Hashtag Activism: Descriptive Findings on Feminist Digital Resistance in Iran Before and After the Mahsa Movement

The selected hashtags were analyzed over two distinct time periods: the pre-Mahsa Amini era (March 2018 to September 2022) and the post-Mahsa Amini period (October 2022 to March 2023). This division highlights the sharp increase in the frequency and symbolic weight of hijab-related hashtags following Amini's death, which catalyzed widespread political unrest across Iran. Prior to this turning point, hashtag activity, particularly those connected to campaigns like White Wednesdays, remained relatively consistent, showing limited fluctuation in intensity or reach.

Since the emergence of the Girls of Enghelab Street, Sneaky Freedoms, and White Wednesdays campaigns, Iranian society has witnessed a visible transformation in women's expression of hijab. A pivotal moment occurred in December 2017, when Vida Movahed—later known as “the Girl of Enghelab Street” stood at the intersection of Enghelab and Vesal Shirazi streets in Tehran, removed her headscarf (Hijab), and waved it atop a stick to protest the compulsory hijab. This symbolic act quickly resonated nationwide. In the following days, similar protests erupted across Tehran and other cities, with images and videos rapidly going viral on social media platforms.

In response, Iranian authorities sought to suppress the visibility of such acts. The police physically modified the public platforms used by protesters, replacing flat surfaces with sloped, triangular structures, to prevent others from standing and reenacting the gesture. According to the Tehran Police Information Center, 29 women were arrested for similar protests between December 27, 2017, and February 1, 2018 (Radio Farda, 2018).

While official statistics on the number of unveiled women remain undisclosed, reports from Iran's key decision-making bodies, including the Islamic Parliament, indicate a growing societal shift. In 2016 and 2017, when these campaigns were gaining momentum, many Iranian women increasingly resisted compulsory Hijab (Khosravi Ooryad, 2024). A study conducted by the Islamic Parliament Research Center from March to September 2018 distinguished between “Islamic hijab” and “customary hijab,” deeming the latter noncompliant with the government's religious standards. According to the study, 60–70% of women in Iran were classified as wearing “bad hijab,” with 10–15% falling into what was described as a serious category, those whose appearance visibly challenged social norms. Additionally, 70% of surveyed women were said to fall into a so-called “grey zone,” although the report did not define this category explicitly (KhabarOnline, 2018).

On the other hand, comparing the content produced in some platforms like Instagram and that generated by news agencies that are against women with no mandatory hijab indicates a continuous conflict between the ruling system and the women who are in favor of optional hijab. For instance, the release of a photo in October 2021, displaying some female students posing for the photo in front of the University of Tehran's main entrance while wearing graduation gowns and no hijab, provoked numerous reactions in the social networks (Ensafnews, 2021). Moreover, a short video, which became viral in June 2022, showed a group of young women with no hijab in Shiraz (7sobh, 2022).

Following widespread support for the hashtag #NoToHijab in June 2022, and the circulation of photographs showing unveiled women in public spaces across various Iranian cities, the campaign gained considerable momentum online. In response, Ahmad Vahidi, who was serving as the Interior Minister of the Islamic Republic at the time, publicly cautioned internet users against being influenced by what he described as a destabilizing campaign (Radio Farda, 2022). Likewise, Masoud Setayeshi, then spokesperson for Iran's judiciary, characterized opposition to mandatory hijab as a foreign-led effort to “spread immorality in society” (BBC Persian, 2022).

According to data from Trendsmap, the hashtag #NoToHijab appeared in over 76,000 tweets globally on July 11 and 12, 2022. On July 12, it was also ranked as the second most popular Persian-language hashtag on the Iranian analytics platform Tagminer.

Some Iranian state-affiliated news agencies framed the protest movement as “an attempt by opposition forces to promote the eradication of hijab and spread promiscuity in Iran.” They also claimed that the associated hashtags failed to trend on Persian-language X (Twitter). Nonetheless, during the 6 months of nationwide protests following the death of Mahsa (Zhina) Amini, many women engaged in public acts of defiance by burning their headscarves, a powerful rejection of the state's mandatory hijab policy. Throughout this period, an increasing number of citizens who did not adhere to the government's definition of appropriate hijab chose to appear unveiled in public spaces (BBC Persian, 2023). As a result, resistance to compulsory veiling gained unprecedented visibility and momentum.

In response, the state intensified its efforts to suppress the movement through various surveillance mechanisms. Police forces employed smart devices and surveillance cameras in public areas and on roadways to identify women who appeared without a hijab. Those identified received formal warnings and notifications outlining the legal consequences of their noncompliance. These measures were designed to deter further acts of civil disobedience by reinforcing the legal risks associated with rejecting the state-imposed dress code.

An analysis of tweets containing hashtags such as #GirlsOfEnghelabStreet, #WhiteWednesdays, #SneakyFreedoms, and #WomanLifeFreedom reveals recurring themes of resistance, empowerment, and rejection of the state's ideological authority. The content often carried powerful and emotionally charged messages, including: “Do whatever you can to knock us down and we will rise higher every time,” “I have a dream which is not impossible to come true,” “Men support women in protesting mandatory hijab,” “We will rise again,” and “We will not go backward.” Other posts articulated the volatile political climate following Mahsa Amini's death, using idioms such as “After Mahsa Amini, everything is tied to one hair,” a metaphor denoting high instability. Additional messages explicitly linked hijab resistance to broader political transformation: “If mandatory hijab is abolished, the rest will be easy,” “Don’t let the regime show its false authority through imposing mandatory hijab,” and “Your hair is the symbol of struggle for freedom.” One widely shared tweet stated: “The more the number of unveiled women, the more difficult it is for the regime to identify them,” a direct reference to state surveillance practices such as facial recognition through traffic cameras.

These expressions reflect more than symbolic protest; they reveal a collective effort to transform imposed objectivity into agentic subjectivity by rejecting the hegemonic definition of hijab. The hashtags function as vehicles through which women publicly challenge the Islamic Republic's political system and its gendered control. More broadly, the discourse embedded in these tweets articulates a rejection of political Islam in its current authoritarian form, casting hijab as one of the central signifiers of the dominant discourse. In this way, the hashtag content not only documents a political moment but also represents a deeper epistemic conflict between institutional authority and emergent, transformative subjectivities.

This process was analyzed using Lacanian codes applied to a stratified sample of 1,000 high-engagement tweets. Tweets were categorized into three themes: those that invoked or negated the master signifier (e.g., “modesty” and “honor”); those that enacted symbolic rupture by destabilizing gendered norms (e.g., “#WomanLifeFreedom is our weapon, not your morality”); and those that demonstrated negation of the Other's desire, such as “Hijab is not my choice, it's your fear.” These tweets functioned not only as protest, but as speech acts—brief interventions that rejected imposed subject positions and reasserted symbolic agency. Approximately 45% of coded tweets referenced the master signifier, 30% enacted symbolic rupture, and 25% expressed subjective negation. By linking digital discourse to symbolic disruption, these tweet patterns illustrate how feminist hashtags become sites of subject formation in a contested ideological space.

Together, these coded tweets reflect not isolated resistance but a collective psycho-discursive formation. The act of tweeting itself, especially under conditions of surveillance and censorship, can be read as a speech act in Lacanian terms: an utterance that breaks imposed silence and asserts presence in symbolic space. Hashtagging becomes not only an act of protest, but also a form of subjectivation, wherein Iranian women reenter the field of discourse not as moral objects, but as political subjects.

Importantly, this psycho-symbolic process intensifies at political flashpoints. Following Mahsa Amini's death, the volume and affective intensity of symbolic ruptures increased sharply. Hashtags such as #WomanLifeFreedom and #IAmAFReeWoman surged not only in frequency but in their capacity to absorb trauma, assert identity, and amplify contradiction. The return of certain hashtags across years, for example, #WhiteWednesdays reemerging in late 2022, also signals a repetition compulsion, where women insistently rearticulate themselves within a discursive field that tries to erase them. In contrast to traditional metrics of political communication, these symbolic forms cannot be reduced to information transfer. Rather, they constitute an ongoing effort to dismantle and rewrite the state's master discourse, one tweet at a time.

From a Lacanian perspective, the repetition and circulation of hashtags within digital protest spaces can be interpreted as a symbolic act through which the subject seeks reentry into the discursive order on her own terms. These acts of digital enunciation, especially those centered on the body, visibility, and defiance, mark the refusal to be fixed as an object of the dominant ideological gaze. The state's imposition of hijab functions as a master signifier, one that seeks to anchor women within a rigid symbolic framework of obedience and moral order. In contrast, the proliferation of tweets declaring agency, desire, and resistance signifies the subject's rearticulation beyond that fixed position. By reappropriating visibility through public declarations such as “I am a free woman” or “My hair is my weapon,” women perform a symbolic negation of the state's control, asserting presence where absence was once demanded. This shift exemplifies what Lacan calls the traversal of the fantasy: a moment when the subject disidentifies with the role assigned by the Other and instead begins to signify herself through her own desire. In Iran's digital protests, this psychic rupture is not only internal but politically generative, as it enables new collectivities and subjectivities to emerge in defiance of systemic objectification.

User engagement with the hashtags #GirlsOfEnghelabStreet and #WhiteWednesdays remained steady throughout the study period, primarily expressed through quoting, retweeting, and personal commentary. While fluctuations in activity were limited, notable peaks occurred: #GirlsOfEnghelabStreet saw its highest frequency between October 2018 and September 2020, whereas #WhiteWednesdays peaked from March 2018 to October 2019. Significantly, the latter experienced a revival after Mahsa Amini's death, with nearly 700 instances recorded between October 2022 and March 2023—demonstrating renewed resonance under shifting sociopolitical conditions.

Additional hashtags such as #OurCameraOurWeapon, #WalkingWithNoHijab, #NoToHijab, #OptionalHijab, and #NoToMandatoryHijab also featured prominently during the observed timeline. Among these, #NoToMandatoryHijab registered the highest volume, with 26,934 tweets between June 2022 and March 2023. A substantial portion—approximately 23,700 posts—was attributed to the Tavaana platform. While its presence prior to mid-2022 was minimal, the sudden surge points to its growing significance within digital feminist discourse. Similarly, #OptionalHijab, first appearing in March 2018, displayed a brief increase in September 2020 and has since followed a consistent upward trajectory, especially from late 2022 onward.

The hashtag #OurCameraOurWeapon showed a notable increase in activity in March 2018, after which its usage plateaued for a prolonged period. However, beginning in June 2022, the hashtag exhibited a renewed upward trend. Following the death of Mahsa Amini in September 2022 and the subsequent nationwide protests, a new wave of hashtags emerged, reflecting a shift in the focus of online activism related to compulsory hijab. These newer hashtags maintained thematic continuity with earlier campaigns but articulated protest demands with increased clarity and urgency. In particular, #WomanLifeFreedom—circulated in both Persian and Kurdish—and #IAmAFreeWoman became prominent expressions of resistance, encapsulating the protestors’ collective defiance of state-imposed gender norms and further intensifying public confrontation with the authorities.

The hashtag #NoToReligiousGovernment also experienced a gradual rise beginning in March 2020. It appeared in over 80 tweets in September 2021 and gained broader traction from September 2022 onward, reflected in increased mentions through tweets, retweets, and opinion posts. Meanwhile, the hashtags #NoToHijab and #NoToObligatoryHijab began appearing more frequently from May 2022 onward. By the end of the study period, they had been generated approximately 20,000 and 3,000 times, respectively, indicating their growing role in the digital discourse on women's autonomy and civil resistance.

**Graph 1.**
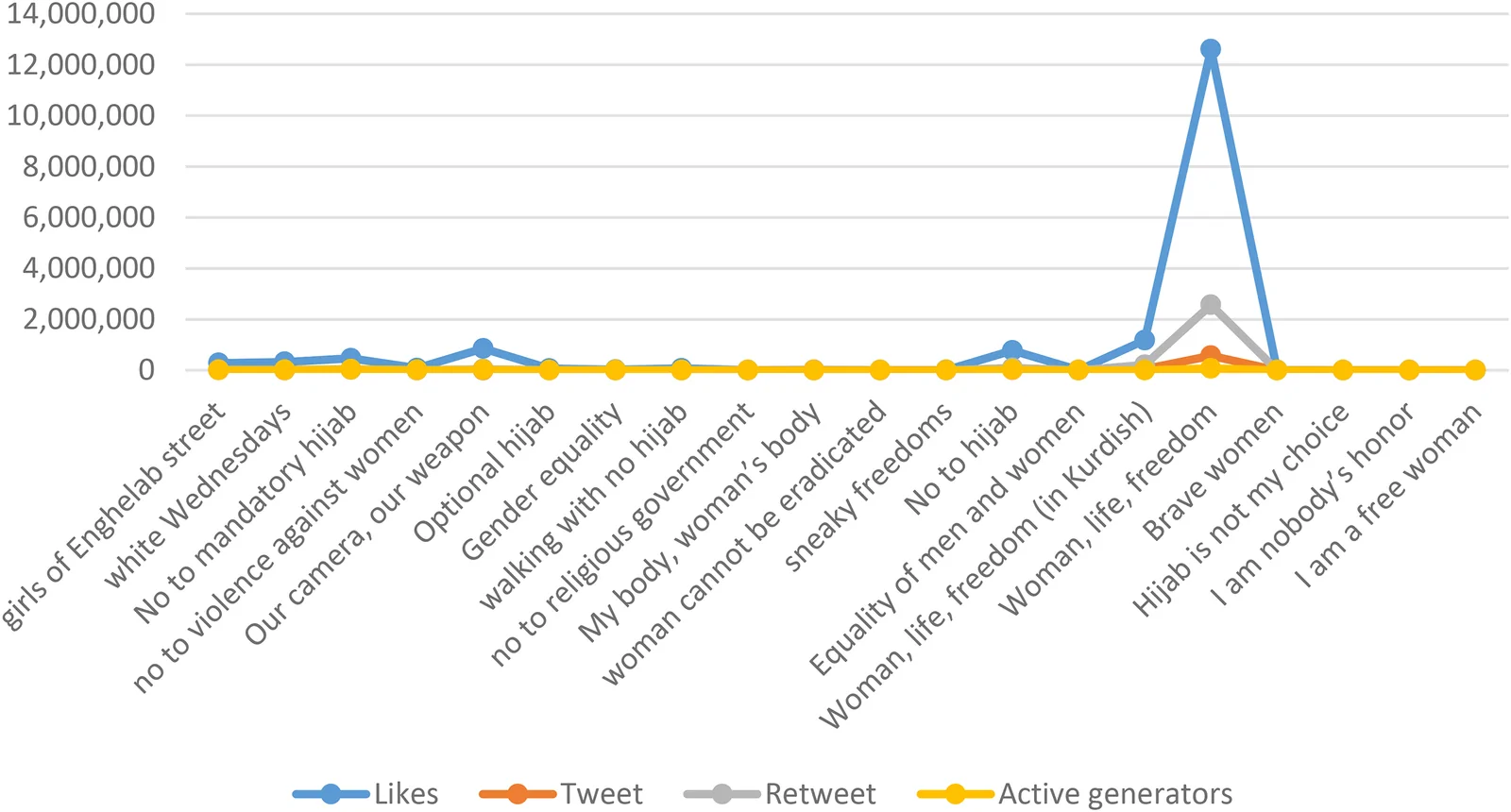
Frequency graph of 20 selected hashtags on X.

Graph 1 provides a frequency analysis of 20 selected hashtags on X, capturing their engagement through metrics such as active generators, retweets, tweets, and likes, as observed in data collected. The hashtag #WomanLifeFreedom recorded the highest engagement across all metrics within this data set, reflecting its symbolic centrality in Iran's contemporary feminist uprising and its broader circulation in transnational solidarity networks (Afary & Anderson, 2023; Izadi & Dryden, 2024).

Following closely, #WomanLifeFreedom in the Kurdish language and #OurCameraIsOurWeapon also exhibit substantial engagement, with the latter showing particularly high likes, reflecting their significance within Kurdish feminist activism and visual advocacy efforts. Meanwhile, #NoToHijab and #NoToMandatoryHijab display notable but comparatively lower engagement, with significant retweets and likes, suggesting a focused but less widespread resonance in discussions around resisting mandatory veiling policies. This variation in engagement underscores the differential impact of these hashtags, highlighting X's role in shaping the visibility of diverse feminist narratives within digital activism spaces.

## Discussion

The interpretive analysis of hashtag trends from 2018 to 2023 reveals more than a chronology of digital protests; it reflects a collective psycho-political rupture in the symbolic order enforced by the state. According to Lacan, subjects are produced through their entry into the symbolic—language, law, and ideology—which is governed by the “Name-of-the-Father,” a metaphor for normative authority. In the Iranian context, the hijab operates as a central signifier of this symbolic order. Resistance to hijab, therefore, is not just disobedience to a dress code, but a rejection of the state's master discourse and its attempt to fix the female subject as modest, obedient, and veiled.

The campaign hashtags examined, particularly #womanlifefreedom, #noToHijab, and #sneakyfreedom, can be seen as signifiers of what Lacan calls the “desire of the subject,” a desire that resists being reduced to the Other's demand. The prominence and virality of these hashtags after Mahsa Amini's death represent a symbolic revolt in which women reposition themselves from objects within the Islamic Republic's moral discourse to desiring subjects asserting their own symbolic coordinates. This is especially visible in the shift from earlier hashtags like #whiteWednesdays, which operated through semisymbolic gestures (e.g., wearing white scarves), to more declarative linguistic markers like #IAmAFreeWoman and #HijabIsNotMyChoice, which openly negate the dominant signifier and assert an alternative identity.

Across the data set, hashtags such as #WomanLifeFreedom, #IAmAFReeWoman, and #HijabIsNotMyChoice did not merely circulate as rhetorical slogans; they operated as master signifiers in flux, attempting to overwrite or destabilize the ideological fixity imposed by the state. For example, tweets repeating “#IAmAFReeWoman and I refuse to disappear” enact a speech act in Lacanian terms, representing a performative rupture that asserts symbolic presence against an erasing authority.

Similarly, emotionally charged phrases like “They fear our joy more than our anger” or “This grief is louder than your morals” reflect jouissance, where pain and defiance are libidinally fused. These are not isolated cases; they emerged repeatedly in many tweet samples, particularly during periods of heightened protest. The systematic coding process captured these textual recurrences, demonstrating that hashtag activism in post-Mahsa Iran produces not only political expression but symbolic friction, through which subjectivity reclaims space in the face of state discourse.

From a Lacanian lens, this transition signifies the subject's movement from the Imaginary and Symbolic into a moment of rupture what Lacan refers to as the Real. The Real is that which cannot be fully captured by discourse, and Mahsa Amini's death functioned as a traumatic event that punctured the symbolic equilibrium, enabling a flood of discursive rearticulations. The hashtag #womanlifefreedom, for instance, functions as a new master signifier, not of submission but of resistance and emancipation, binding together fragmented acts of digital disobedience into a coherent oppositional discourse (Beidollahkhani & Farkhari, 2024). This mirrors Lacan's notion of how subjects create new symbolic orders by forging new chains of signifiers.

Additionally, the temporality of hashtag emergence and repetition reflects the compulsive return of the subject's desire. The repeated circulation of slogans across months and years illustrates a collective attempt to “write” a new subjectivity into the social field. This process is not linear or complete; it echoes Lacan's theory of the barred subject (S/), whose identity is always in flux and shaped through lack. Iranian women's digital expressions are shaped by this fundamental lack, what is denied to them in law and society, and it is precisely through this lack that resistance takes root.

Moreover, engagement with X (Twitter), despite its status as a banned platform in Iran, demonstrates a paradox in which the state imposes silence while digital tools facilitate the emergence of a counter-symbolic field. The symbolic act of tweeting itself can be interpreted as a transgressive utterance, what Lacan would call a “speech act” that breaks the cycle of imposed passivity. This speech act becomes a process of subjectification: the user is no longer merely the object of the master's gaze but becomes the enunciator of a new narrative.

Finally, while hashtags operate as linguistic devices, they also function as affective machines. They are saturated with trauma (#MahsaAmini), irony (#sneakyfreedom), defiance (#NoToHijab), and hope (#WomanLifeFreedom). These effects, embedded in the signifiers, reveal what Lacan described as jouissance, excessive affective investment that disrupts normative discourse. The intense emotional tone surrounding these digital artifacts helps us grasp how the symbolic disruption is not only political but also libidinal and psychic.

In short, by interpreting hashtag activism through a Lacanian lens, we see how Iranian women's digital resistance is not just a political strategy but a psycho-symbolic struggle for self-definition, agency, and reentry into the symbolic field on their own terms.

## Conclusion

This study has explored the evolution and significance of hashtag activism in Iran's feminist resistance, focusing on how women have used X (Twitter) as a platform for digital protest against mandatory hijab and broader state control. Drawing on a data set of 20 influential hashtags from 2018 to 2023, including #WhiteWednesdays, #GirlsOfEnghelabStreet, #WomanLifeFreedom, and #NoToMandatoryHijab, the findings illustrate how Iranian women have turned digital platforms into sustained arenas of dissent. These campaigns have extended far beyond symbolic protest, generating real socio-political pressure and reconfiguring the boundaries of state-society discourse in Iran.

While earlier activism around hijab often relied on physical presence and small-scale disobedience, the post-Mahsa Amini era has seen a sharp transformation. X (Twitter), though filtered in Iran, became a powerful site for women's voices, amplified by diaspora actors and networked influencers. Hashtags have functioned not only as organizational tools but also as affective signifiers of solidarity, resistance, and collective identity formation, contributing to the emergence of new subjectivities among Iranian women as they assert their rights and challenge dominant narratives. This digital activism catalyzed a broader feminist movement that resists the state's ideological project and asserts alternative visions of gender, freedom, and governance.

The study's integration of summative and conceptual content analysis offers both macro and microlevel insights. Quantitatively, it reveals which hashtags gained traction and when; qualitatively, it uncovers how women engaged these digital tools to challenge the state's monopoly on symbolic order. While informed by Lacanian psychoanalysis, particularly the concepts of subjectivity and symbolic rupture, this paper centers the activists themselves, their strategies, voices, and networks, as the driving force behind change.

The implications are both local and global. In authoritarian contexts where public dissent is criminalized and women's bodies are heavily policed, social media becomes a vital space for visibility and resistance. Iranian women's hashtag activism demonstrates how digital tactics can challenge patriarchal state structures and inspire offline mobilization. It also points to the necessity of transnational solidarity and the importance of documenting, archiving, and analyzing such resistance.

Looking forward, future research should examine how hashtag activism intersects with other digital platforms (e.g., Instagram and Telegram), how state actors adapt and respond to digital dissent, and how these online movements shape long-term legal, social, and cultural change. Scholars must also explore the psychological toll and ethical risks faced by women who participate in such resistance under constant surveillance.

This paper shows that Iranian women's hashtag activism is not a fleeting trend but part of a sustained feminist movement, a movement that resists through data, symbols, images, and words, all woven into a digital fabric of defiance. In reclaiming their digital space, Iranian women are not only protesting against the hijab; they are demanding a reimagination of society itself.

In sum, Iranian women's hashtag activism represents a powerful form of political resistance and identity reclamation. It is not only transforming public discourse on gender and governance within Iran but also contributing to a global understanding of how digital technologies can empower feminist struggles in even the most restrictive political setting.

## Notes

1. Masih Alinejad, an Iranian-American journalist and women's rights activist, is widely recognized for her outspoken critique of human rights abuses in Iran. She has played a significant role at Voice of America's Persian News Network and has contributed to Radio Farda and IranWire. Alinejad founded the My Stealthy Freedom movement, which advocates against forced Hijab laws. Her efforts have earned her numerous accolades, including recognition as one of Time magazine's 2023 Women of the Year.

2. Tavaana E-Learning Institute is an online educational platform dedicated to empowering Iranian civil society. It offers free access to a wide range of training resources and courses in both Persian and English, aimed at fostering personal and professional development. Launched in 2010, Tavaana, derived from the Persian word for “empowered” and “capable” is committed to promoting active citizenship and strengthening civic leadership in Iran through education and capacity-building initiatives.

3. Ali Karimi is an Iranian football player who formerly played for the national team and was recognized as one of the best players in Iran and Asia. After Mahsa Amini's death in September 2022, he became a vocal critic of the Islamic Republic's political system and increased his activities in the virtual space. Eventually, he left Iran and continued to oppose the ruling regime.

4. Iran International is a Persian-language satellite television network, radio channel, and news website based in London, dedicated to broadcasting news and political and social programs concerning Iran. The Iranian government perceives this network as a “sinister” entity, allegedly established by Saudi Arabia, its primary regional rival in the Middle East, with the purported intent of undermining Iran's governmental authority.

5. A former reporter and expert with BBC Persian has been recognized as a media activist advocating for the overthrow of the Islamic Republic. Currently operating as a freelance journalist and social media activist on X, this person has contributed numerous articles critical of the Islamic Republic across various media platforms in recent years.

6. Seyyed Mostafa Tajzadeh, a notable Iranian reformist politician and former deputy interior minister, has been a significant figure in advocating for political change within Iran. He was convicted on charges of assembly and collusion against national security, as well as propaganda activities against the regime.

7. Manoto is an international Persian-language satellite television channel, established in October 2010 by Keyvan and Marjan Abbasi, with its headquarters originally based in London. However, due to financial constraints and political pressures, the channel ceased its broadcast operations in January 2023. It has since shifted to maintaining an active online presence, primarily through platforms such as YouTube and other social media channels, where it continues to engage its audience with Persian-language content.

8. IranWire is an independent news platform founded in 2013 by Iranian-Canadian journalist Maziar Bahari. It combines professional and citizen journalism to report on Iranian affairs, focusing on human rights, civil society, and freedom of expression. The site publishes in Persian and English, providing a forum for Iranians inside and outside the country to share news and perspectives.
